# TP63 regulates pyroptosis via NF-κB/NLRP3/caspase-1 signaling in interstitial cystitis: insights from single-cell sequencing

**DOI:** 10.1515/med-2026-1476

**Published:** 2026-07-31

**Authors:** Wantong Xue, Minli Shi, Lei Pang

**Affiliations:** Department of Urology, The Fifth Hospital of Shanxi Medical University (Shanxi Provincial People’s Hospital), Taiyuan City, Shanxi Province, China

**Keywords:** interstitial cystitis, single cell sequencing, bioinformatics analysis, cell pyroptosis, TP63

## Abstract

**Objectives:**

This study aims to explore the regulatory role of the TP63 gene in interstitial cystitis (IC) and its molecular mechanism of involvement in disease occurrence and development through the pyroptosis pathway through single-cell sequencing and bioinformatics analysis, and evaluate its potential as a therapeutic target and biomarker.

**Methods:**

A 6–8 week SPF grade female SD rat IC model was constructed by intraperitoneal injection of cyclophosphamide (n=10/group, repeated 3 times). The model construction effect was verified by the Von Frey fiber tenderness method, urodynamic evaluation, and bladder histopathological analysis. Screening key genes related to apoptosis in bladder tissue of IC rats using single-cell sequencing and bioinformatics analysis. *In vitro* experiments were conducted using lipopolysaccharide (LPS) to treat immortalized human ureteral epithelial cells (SV-HUC-1) to establish a cellular inflammation model (n=6/group, repeated 3 times), and the transcription level changes of the TP63 gene were detected by RT-PCR. Western blotting was used to detect the expression of apoptosis-related proteins and NF-κB/NLRP3/caspase-1 signaling pathway proteins (caspase-1, GSDMD, ASC) in the bladder tissue of IC rats. The CCK8 method and EdU experiment were used to evaluate cell survival rate and proliferation ability, respectively. ChIP-qPCR was conducted to detect the binding of TP63 to the RELA promoter region. The role of caspase-1 in the pyroptosis pathway was verified using the caspase-1 inhibitor.

**Results:**

Single-cell sequencing and bioinformatics analysis showed that the TP63 gene was significantly upregulated in the bladder tissue of IC rats (p<0.0001). Western blotting results showed a significant increase in the expression of apoptosis-related proteins in the IC model (p<0.0001), while HE staining showed a significant increase in the infiltration of mast cells, macrophages, and T cells (p<0.05). *In vitro* experiments, Western blotting results showed that after LPS intervention, the expression of NLRP3 inflammasome component (ASC) and downstream effector molecule (GSDMD) in SV-HUC-1 increased, the apoptotic characteristic protein caspase-1 was activated, CCK-8 detected a decrease in cell viability, and Edu experiments showed inhibition of cell proliferation. After TP63 siRNA intervention, all the above effects were reversed. ChIP-qPCR confirmed that TP63 directly binds to the RELA promoter region, activating NF-κB signaling. Furthermore, caspase-1 inhibition experiments demonstrated that suppressing caspase-1 significantly alleviated TP63-mediated pyroptosis and inflammatory responses.

**Conclusions:**

TP63 contributes to the pathogenesis of IC by directly binding to the RELA promoter, activating NF-κB signaling, and subsequently regulating the NLRP3/caspase-1/GSDMD axis to induce bladder epithelial cell pyroptosis. TP63 shows promise as a potential biomarker and therapeutic target for IC.

## Introduction

Interstitial cystitis (IC) is a bladder dysfunction characterized by frequent urination, urgency, and pain and discomfort in the bladder or pelvis [1]. Epidemiological data show that there are significant regional differences in the prevalence of IC in the world (0.1–4% in Europe and the United States, and about 0.01–0.5 % in Asia), and there is a significant gender imbalance (the incidence rate of women is 5–10 times that of men). The high incidence age is concentrated in 30–50-year-olds [2], 3]. It is worth noting that due to inconsistent diagnostic criteria (excluding other causes such as infection) and overlapping symptoms with urinary system diseases (such as overactive bladder), about 40 % of patients take more than 5 years to be diagnosed, and the actual incidence rate may be seriously underestimated. IC, as a progressive disease, has a long and recurrent course, with about 70 % of patients reporting worsening symptoms over time [4]. This condition not only increases annual per capita medical costs by 2- to 3-fold (from approximately $4,200 to $8,500–12,600), but also causes more than 50 % of patients to develop comorbidities such as anxiety/depression [5].

The current treatment dilemma of IC mainly stems from its complex pathogenesis. Despite various hypotheses such as mast cell activation, epithelial barrier disruption, and neuroinflammation [6], there is no single theory that can fully explain its clinical heterogeneity. Among them, bladder epithelial dysfunction is considered a core link: on the one hand, the loss of tight junction proteins (such as ZO-1, occludin) leads to the infiltration of potassium ions and other substances in urine, directly activating submucosal nociceptive nerve fibers; On the other hand, mediators such as ATP and substance P released by damaged epithelium drive neuroimmune crosstalk through purinergic receptors and NK1 receptors, forming a vicious cycle of pain inflammation [7]. In recent years, studies have found that bladder epithelium may participate in the pathological process of IC through various programmed cell death mechanisms, especially cell pyroptosis – an inflammatory death mediated by NLRP3 inflammasomes that shows characteristic activation in bladder biopsy tissues of IC patients: the expression level of pyroptosis executing protein GSDMD is 3–5 times higher than that of the control group, and is positively correlated with pain scores (r=0.62, p<0.01) [8]. Studies have shown [9] that induction of the IC model by cyclophosphamide confirms that the inflammatory cytokine IL-1β level in bladder tissue of NLRP3 knockout mice decreases by 60 % and mast cell infiltration decreases by 45 %, providing a theoretical basis for targeting the apoptotic pathway.

The TP63 gene, as a member of the p53 family, plays a crucial role in the development and maintenance of urinary system homeostasis. Research has shown that: ①The bladder specific Δ Np63αsubtype is the main regulatory factor for urinary tract epithelial differentiation, and its absence can lead to the destruction of epithelial barrier integrity; ②In a chronic inflammatory environment, TP63 can regulate the secretion of pro-inflammatory factors such as IL-6 and IL-8 through the NF – κB pathway; ③The latest single-cell sequencing has found abnormal expression patterns of TP63 in bladder epithelium of IC patients, suggesting its possible involvement in disease-specific transcriptional reprogramming [10]. However, whether TP63 affects IC progression by regulating cell apoptosis is currently a knowledge gap.

Based on the above evidence, we propose a scientific hypothesis: TP63 regulates NLRP3/GSDMD-mediated apoptosis of bladder epithelial cells, driving inflammation amplification and tissue damage in IC. This study will integrate single-cell transcriptome analysis and functional experimental validation, aiming to: ①establish the molecular association between TP63 and the pyroptosis pathway in IC; ②Elucidate the specific mechanism by which TP63 regulates pyroptosis; ③Evaluate its translational potential as a therapeutic target. The research results not only provide a new basis for the molecular typing of IC but also have the potential to open up new avenues for the development of precise therapeutic strategies targeting epithelial repair.

## Materials and methods

### Experimental animals

Based on epidemiological statistics, we selected 20 SPF-grade female SD rats, aged 6–8 weeks, with a body weight of 180 ± 20 g, as experimental subjects. The feeding environment was supported by the Animal Experiment Center of Shanxi Provincial People’s Hospital, and all experimental rats were housed in the center’s SPF grade non-pathogenic animal facility, with room temperature maintained at 20 °C–26°Cand humidity maintained at (50 ± 20)%. Based on preliminary experimental data and literature reports, we used G*Power software (version 3.1) to estimate the sample size. With a statistical power (1-β) of 0.8, α=0.05, and an effect size (d) = 1.5, the calculation indicated a minimum requirement of 8 rats per group. To ensure sufficient reliability of the experimental results and statistical power, the final sample size was set to 10 rats per group in this study.

### Animal model construction and validation experiments

This experiment selected healthy adult female SD rats as experimental subjects, calculated the dose of cyclophosphamide (CYP) based on body weight (25 mg/kg), and established a CYP group and a control group. The CYP group rats received intraperitoneal injections of CYP on days 1, 3, 5, 7, and 9, while the control group was given an equal volume of physiological saline. The experiment strictly follows aseptic procedures, with the CYP solution preheated in advance and slowly injected to reduce stress reactions during injection.

#### Von Frey fiber tenderness method

Nine levels of Von Frey fiber filaments (1.0 g, 1.4 g, 2.0 g, 4.0 g, 6.0 g, 8.0 g, 10.0 g, 15.0 g, 26.0 g) were used to mechanically stimulate the abdomen of rats and evaluate pain response. Prior to testing, each rat’s designated abdominal area was prepared with skin, and the animals were placed in transparent Plexiglas boxes, placed on raised mesh floors, and allowed to acclimate for at least 30 min. During the implementation of the Von Frey test, each fiber filament was stimulated on the abdomen of rats through a grid with a force that could slightly bend its shape for 1–2 s. Each fiber was repeated 3 times, with a 5-s interval between each stimulation, and the stimulation area near the bladder was carefully shifted to avoid desensitization. The pain response score is as follows: 0=no response; 1=Abdominal contraction; 2=Contraction plus positional change; 3=Contraction, positional change, stimulation area licking, and/or vocalization. The pain score of rats is determined based on their maximum pain response score exhibited in three stimuli.

#### Urinary motility assessment

Before starting the experiment, it is necessary to exhaust air from the infusion pump and pump tube, and set the pump to 6 mL/h. On the rat platform, place the rat in a supine position, induce anesthesia by inhaling isoflurane, and maintain it through an animal mask. Disinfect the urethral opening, then insert a 19-G pressure-measuring catheter into the bladder 3 cm deep through the urethra and secure it with tape. By administering physiological saline infusion, monitoring urodynamic indicators using computer software, and recording data using a pressure gauge, the experiment begins when the urodynamic curve stabilizes.

#### Pathology of bladder tissue

After euthanizing the animals, the bladder was quickly collected and evaluated for bladder quality, wall thickness, and edema. The bladder is fixed in a 10 % (volume fraction) formaldehyde solution and embedded in paraffin. Bladder slices were stained with hematoxylin and eosin (HE). According to the standards established by Gray et al., macroscopic examination was performed on each bladder to evaluate edema. No, mild, moderate, and severe edema were rated as 0/1/2/3 points, respectively. When fluid is visible on both the exterior and interior of the bladder wall, it is defined as severe edema; When edema is limited to the internal mucosa, it is defined as moderate edema; When edema is between normal and moderate, it is defined as mild edema.

### Single-cell sequencing analysis

#### Preparation of bladder single-cell suspension

Under sterile conditions, the CYP group and control group rats were euthanized using the cervical dislocation method, and the bladder tissue was removed and cut into pieces. Centrifuge the tissue fragments, remove the supernatant, and then blow and centrifuge with DPBS. Add collagenase I and DNase I to digest the tissue, centrifuge to remove the supernatant, and then digest with StemPro Accutase. Terminate digestion with DPBS, centrifuge to remove supernatant, add red blood cell lysis buffer to lyse red blood cells, and centrifuge again to remove supernatant. Wash the cell suspension with DPBS, centrifuge to remove the supernatant, and repeat once. Obtain bladder single-cell suspension by centrifugation through a 40 μm cell sieve, resuspend with DPBS, and transfer into an EP tube. Mix 10 μL of cell suspension with trypan blue, count under a microscope, and calculate the cell survival rate. The cell survival rate should be greater than 80 %. Enrich live single cells and load them for single-cell sequencing.

#### scRNA seq of bladder single cells

The living cells are combined with gel bead suspension containing Barcode and oil drops in Chromium Chip B to form gel beads (GEMs) through microfluidics. After GEMs were collected, gel beads released Barcode sequences and cells released mRNA. mRNA is converted into cDNA with a barcode and UMI by reverse transcriptase, and then subjected to SMART amplification. Finally, the cDNA was fragmented, purified, and amplified by PCR. After screening the fragments, a cDNA library with P5 and P7 adapters was constructed. After the library is constructed, it undergoes library inspection, and qualified data is sequenced using an Illumina sequencer for post-processing.

#### Data quality control

The scRNA data analysis was conducted using the 10 × Genomics platform. This platform has the advantages of high cell flux, short cycle, and high single-cell capture efficiency, and has become a powerful tool for identifying cell heterogeneity, discovering new cells, analyzing cell development trajectories, and screening antibodies [11]. Use the official Genomics software CellRanger ARC to perform quality control and filtering on experimental data (feature value>300; Count value<100,000; Mitochondrial gene content<10 %), and exclude data from multicellular, bicellular, or unbound low-quality cells.

#### Dimensionality reduction clustering

After controlling for average expression and dispersion relationships, all highly variable genes (HVGs) in single cells were screened, and batch effect correction was performed using Harmony (version 0.1.0). The highly variable genes ranked in the top 2000 after data correction were subjected to principal component analysis (PCA) algorithm for dimensionality reduction, followed by second-order dimensionality reduction and gene expression visualization using uniform manifold approximation and projection (UMAP) algorithm to obtain segmented cell populations.

#### Cell annotation

Set the resolution to 0.3 and use the “findclusters” function to cluster cells into 25 different cell clusters (cluster numbers 0 to 24). Use the FindAllMarkers function to identify the specific marker genes significantly expressed in each cell cluster. Based on the differential gene expression of each cell cluster, refer to the single-cell expression profile dataset for cell type identification. Then, use the SingleR package (V2.2.0) and manually annotate the cell clusters with known marker genes.

#### Analysis of intercellular communication

Using the R software package CellChatDB, based on the differences in ligand and receptor gene expression in different cell types, reveals the communication network relationships between cells. Based on the prediction of cellular communication, further analysis is conducted on the potential of ligand receptor regulation in pathway-related ligand receptor cells, taking into account changes in the expression levels of ligands and receptors in these cells.

#### Differential gene screening

Use the R package DESeq2 (version: 1.44.0) to analyze single-cell sequencing data of the CYP group and control group rat bladder tissue samples, in order to identify differentially expressed genes between IC and normal bladder tissue. Set the threshold for differential expression analysis to | log2FC |>1 and adjust for p<0.05. Upregulated genes satisfy log2FC>1 and p<0.05, while downregulated genes satisfy log2FC<−1 and p<0.05.

#### Functional enrichment analysis

Perform GO and KEGG enrichment analysis on differentially expressed genes (DEGs) to clarify the molecular processes and important pathways that genes may be involved in. Using the GO database, classify and annotate genes and gene products from three aspects: biological processes, molecular functions, and cellular components; Perform pathway analysis on differentially encoded genes using the KEGG database (combined with KEGG annotation results). Use the R language ClusterProfiler package to convert gene IDs into the “ENTREZID” format, and set the GO and KEGG entries for annotated genes to 10. Meanwhile, set P and Q to 0.05 as the criteria for significant enrichment analysis.

#### Core gene screening

By summarizing published literature on cell apoptosis, 27 genes related to cell apoptosis were identified. Through Venn2.1.0 online platform (https://bioinfogp.cnb.csic.es/tools/venny/), the key hub gene TP63 of IC was identified by combining upregulated genes and apoptosis-related genes.

### Cell culture

Cultivate human ureteral epithelial immortalized cells (SV-HUC-1) in DMEM medium (containing 1 % penicillin, streptomycin, and 10 % fetal bovine serum) and place them in an incubator (5 % CO_2_, 37 °C) for further experiments when the cell density reaches around 80 %.

### Construction and validation experiments of the cellular inflammation model

Randomly divide the cells into a control group and an LPS group, with the control group cells cultured normally. The LPS group treated cells with 20 μ g · mL-1 lipopolysaccharide (LPS) for 24 h to establish an *in vitro* model of ureteral epithelial cell inflammation.

#### RT-PCR detection of mRNA expression

Total RNA was extracted from LPS group and control group cells using the Trizol method, and its purity was determined by measuring OD260/OD280. The integrity of the 28 and 18s ribosomal RNA bands was evaluated by RNA electrophoresis, and a PCR reaction mixture was prepared. The reverse transcription PCR reaction mixture was centrifuged in a microcentrifuge. After completion, continue to react in the PCR instrument. During this operation, the reaction conditions need to be controlled at 37 °C×15 min→85 °C×5 s. After the above steps are completed, measure the concentration and purity of the cDNA and transfer it to a low-temperature environment for storage. Each experiment was repeated three times.

#### Western blotting for detecting the expression of related proteins

Collect LPS group and control group cells, extract proteins, and use the BCA assay kit to detect protein content and balance. Then proceed with gelation, sample loading, electrophoresis, and membrane transfer, followed by sealing with 5 % skim milk powder. Subsequently, the primary antibody was treated overnight, and the secondary antibody was incubated at room temperature for 90 min. After washing three times, add the ECL luminescent solution for exposure. Finally, ImageJ software was used for band analysis and data acquisition to obtain the relative expression level of the target protein, with each experiment repeated three times.

### Cytological experimental verification

#### CCK8 method for detecting cell survival rate

Collect logarithmic growth cells from the LPS group and the control group, and inoculate them into 96-well plates after digestion and centrifugation, with the density adjusted to 5,000 cells/well. Add 10 μL of CCK-8 solution to each well at 0 (i.e., after cell adhesion) and 24 h, and incubate in the incubator for 4 h. Use an enzyme-linked immunosorbent assay (ELISA) reader to detect the absorbance (A) value of cells at a 450 nm wavelength and record it. Calculate the cell viability of each group according to the formula: cell viability × (%)=[A (dosing) - A (blank)]/[A (0 dosing) – A (blank)] × 100, and repeat the experiment three times for each group.

#### EdU experiment to detect cell proliferation

According to the instructions of the Edu detection kit, the LPS group and control group logarithmic growth phase cells were seeded in culture plates, incubated with EdU reagent for 2 h, then fixed with 4 % paraformaldehyde, permeabilized with permeabilizing solution for 15 min, removed permeabilizing solution, added Click reaction solution and incubated at room temperature in the dark for 30 min. Hoechst 33,342 reagent was added for nuclear staining for 30 min, and the cell staining was observed and counted using a fluorescence microscope (red fluorescence was Edu staining, blue fluorescence was nuclear staining). Each experiment was repeated three times.

#### Cell scratch detection and cell migration ability

**Figure 1:**
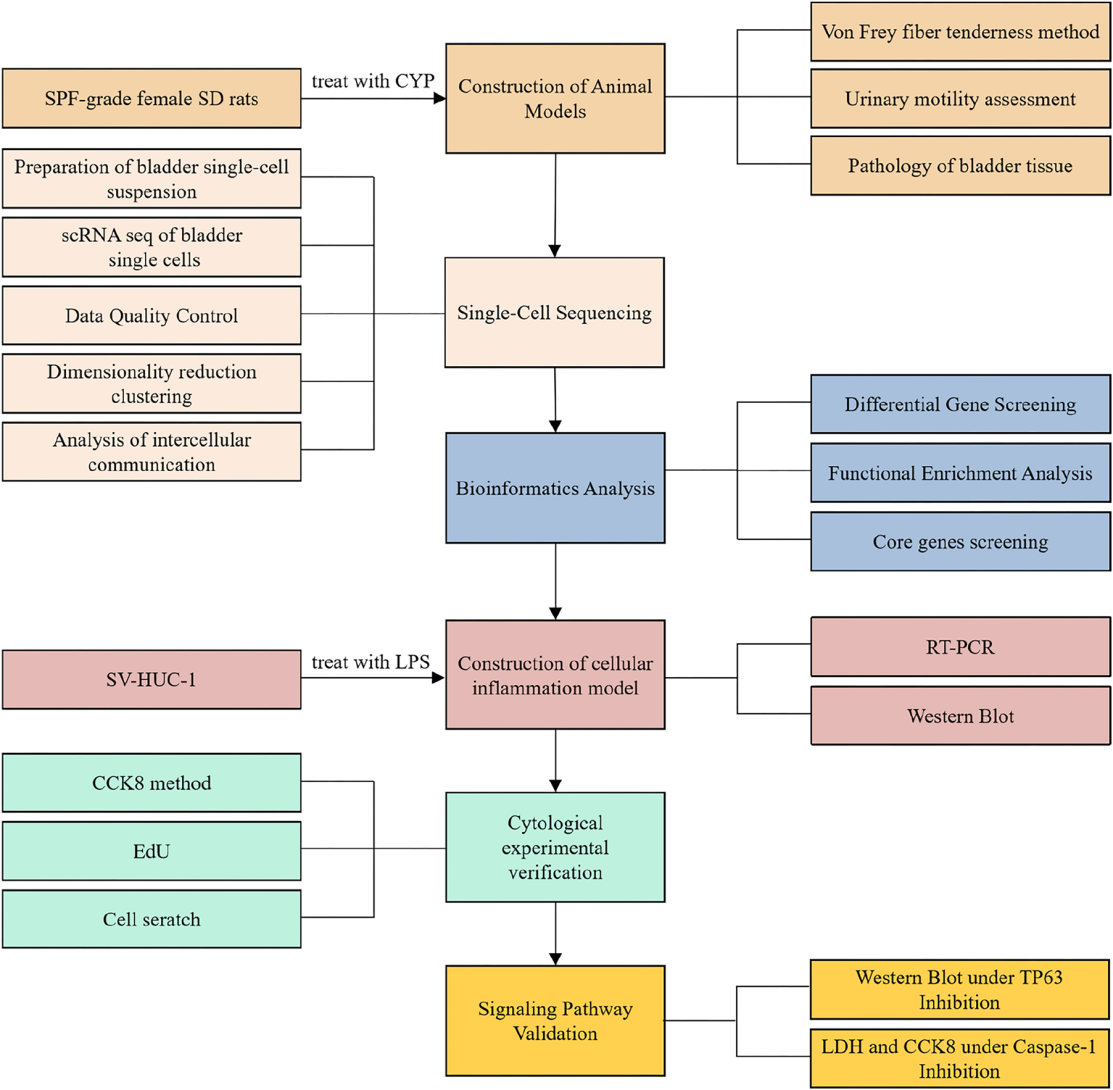
The technical roadmap.

Inoculate the LPS group and control group cells in logarithmic growth phase at a concentration of 5 × 10^5^ cells/well into a 6-well cell culture plate for further cultivation. When the cell density reaches over 90 %, draw three equidistant parallel lines on each well with a 200 μL pipette tip, wash with PBS, and incubate with freshly prepared serum-free medium. Cultivate the cells in a 37 °C 5 % CO_2_ incubator. Record the scratch conditions at 0 and 48 h under an optical microscope, and repeat each experiment three times. The technical roadmap is shown in Figure 1.

### Signal pathway mechanism validation experiment

#### Chromatin immunoprecipitation (ChIP)-qPCR

The RELA gene encodes the core transcriptional subunit p65 of the NF-κB pathway. To investigate whether TP63 activates the NF-κB pathway by directly regulating RELA transcription, we performed chromatin immunoprecipitation followed by quantitative PCR (ChIP-qPCR). Normally cultured SV-HUC-1 cells were used as the control group, while cells treated with 1 μg/mL LPS for 24 h served as the LPS-induced group. Cells were cross-linked with 1 % formaldehyde for 10 min, and the reaction was terminated with glycine. Chromatin was extracted using a cell lysis buffer and fragmented to 200–500 bp using an ultrasonic disruptor. Immunoprecipitation was performed using a TP63-specific antibody, with IgG serving as a negative control. The purified DNA fragments were quantitatively analyzed by SYBR Green qPCR with primers designed for the RELA promoter region. Each experiment included three technical replicates and was independently repeated three times (n=3). Results were calculated as fold enrichment using the Input percentage method and subjected to statistical analysis.

#### Caspase-1 inhibition experiment

To clarify the mechanism by which TP63 regulates pyroptosis via the caspase-1 pathway, the following six experimental groups were compared.

(1) Control group: normally cultured SV-HUC-1 cells
(2) LPS group: treated with 1 μg/mL LPS for 24 h
(3) LPS + siTP63 group: transfected with TP63-specific siRNA followed by LPS stimulation
(4) LPS + caspase-1 inhibitor group: pretreated with 50 μM VX-765 for 2 h, then co-treated with LPS
(5) LPS + siTP63 + caspase-1 inhibitor group: simultaneously subjected to TP63 knockdown and caspase-1 inhibition followed by LPS stimulation
(6) Caspase-1 inhibitor alone group: treated only with 50 μM VX-765 for 24 h

Pyroptosis levels were detected using the LDH release assay. Briefly, culture supernatants from each group were collected, and the working solution was prepared according to the LDH detection kit instructions. The supernatant was incubated with the working solution at room temperature for 30 min in the dark, and the absorbance was measured at 490 nm. The cytotoxicity percentage was calculated based on the formula.

Cell viability was measured using the CCK-8 assay. After treatment, a 10 % CCK-8 solution was added to each well and incubated at 37 °C for 2 h. The absorbance was measured at 450 nm, and the relative cell viability of each group was calculated using the control group as the baseline.

All experiments were performed with six technical replicates and independently repeated three times (n=3). Data are presented as mean±standard deviation, and group comparisons were analyzed using one-way ANOVA, with p<0.05 considered statistically significant.

### Statistical analysis

Statistical analyses were performed using SPSS 26.0 and R 4.2.0. Single-cell RNA sequencing data were analyzed with the DESeq2 package (version 1.44.0), whose negative binomial distribution model is suitable for single-cell data characteristics. Differentially expressed genes were identified using the thresholds |log2FC|>1 and FDR<0.05.

Linear mixed-effects models were applied to repeated measures data, with experimental batches included as random effects. Measurement data are presented as mean ± standard deviation. Group comparisons were performed using t-tests or ANOVA. A p value<0.05 was considered statistically significant. All figure legends specify sample sizes, error bar types, and statistical methods.

### Ethical approval

This experimental protocol has been approved by the Ethics and Welfare Committee of Experimental Animals of Shanxi Provincial People’s Hospital (2021), Provincial Medical Ethics Review No. 154.

## Results

### Successful construction of animal model

#### Von Frey fiber tenderness test results

**Figure 2:**
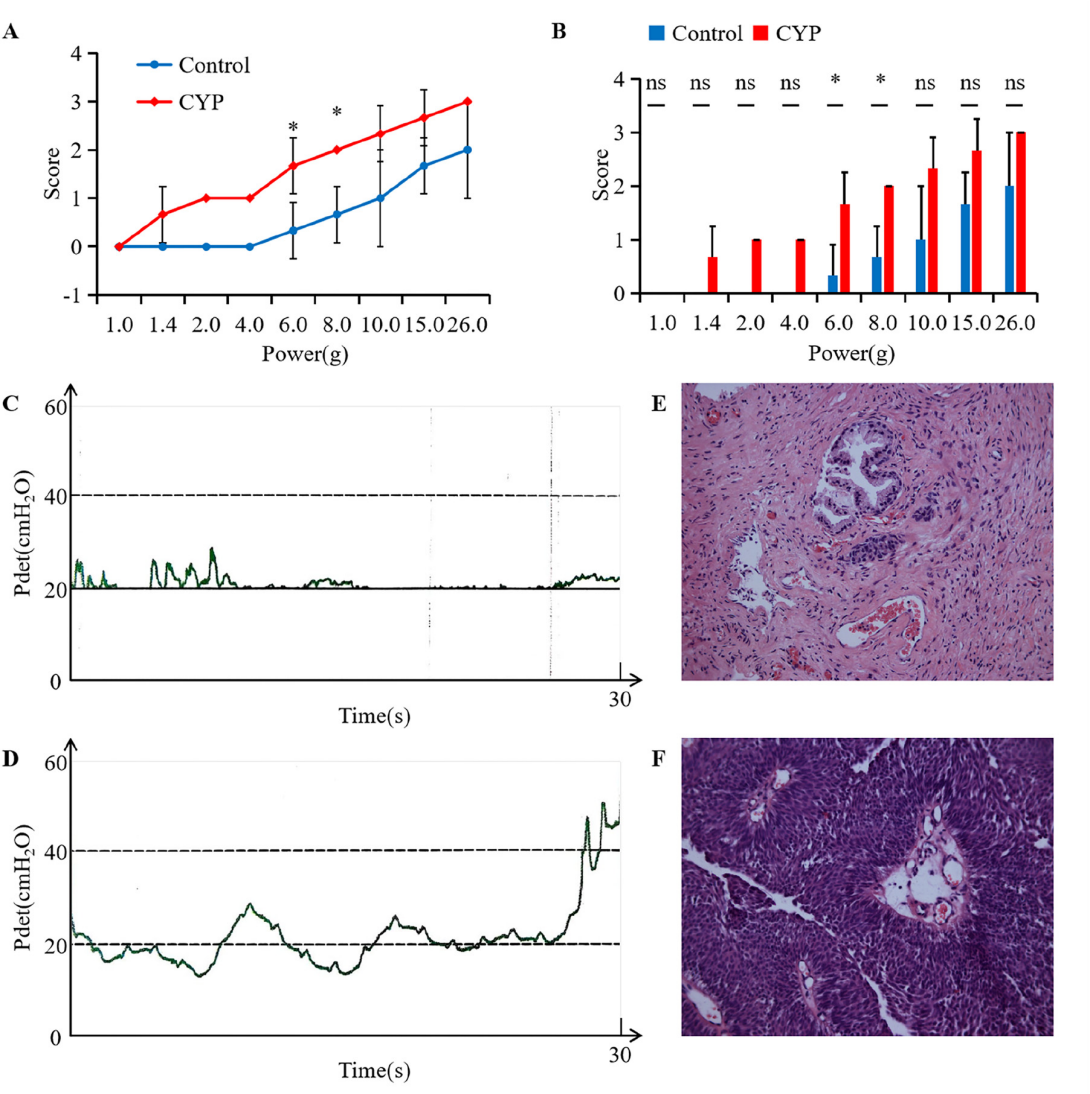
Von frey fiber tenderness, urodynamics, and HE staining results. Note: (A, B) behavioral pain response scores of control (n=10) and CYP (n=10) rats to mechanical stimulation with different von frey filaments. Data are presented as *M*±SD *p<0.05, compared to the control group by independent samples t-test. (C, D) Representative urodynamic traces showing bladder detrusor pressure in control and CYP-treated rats. (E, F) Representative photomicrographs of hematoxylin and eosin (HE)-stained bladder tissue sections from control and CYP rats.

Firstly, we conducted tenderness experiments on the CYP group and the control group rats separately. During the construction of the model, no rat deaths were observed. Forty-eight hours after the completion of model construction, evaluation was conducted through Von Frey fiber filament tenderness experiments. The results showed that intraperitoneal injection of CYP caused pelvic pain in rats, manifested as an increase in response to 6–8 g Von Frey fiber force that was harmless to normal (abnormal pain) (p<0.05) (Figure 2A and B).

#### Results of urodynamic assessment

During the urodynamic evaluation, significant differences were found in the urodynamic indicators between the CYP group and the control group rats (Table 1) (Figure 2C and D). Compared with the control group, the urinary interval of the CYP group rats was significantly shortened (p<0.001), and the maximum detrusor pressure was significantly increased (p<0.001).

**Table 1:** Differences in urodynamics between control and CYP groups.

Dimension (math.)	Control group (n=10)	CYP group (n=10)	*t*	p-Value
	*M* ± SD	*M* ± SD		
Micturition interval	111.57 ± 18.50	38.73 ± 8.57	12.09^a^	<0.0001
Maximum urethral pressure	11.93 ± 1.74	51.58 ± 11.03	12.05^a^	<0.0001

Data are shown a mean ± standard deviation (*M* ± SD)*.*“a” indicates p<0.0001 compared with the control group. A significant difference between groups was determined by an independent samples t-test.

#### Pathological examination results of the bladder

The histopathological results are shown in Figure 2E and F. Observation under an optical microscope revealed bladder inflammation in the CYP group rats, presenting severe interstitial edema, significant infiltration of inflammatory cells, edema of the lamina propria, submucosal bleeding, and focal damage to the urinary tract epithelium; On the contrary, the bladder tissue of the control group showed no signs of inflammation and minimal infiltration of mast cells, which once again confirms that inflammation and immune response are key features of IC [12].

The above results demonstrate the successful construction of the model.

### Single-cell sequencing identifies TP63 as a key gene regulating pyroptosis

#### scRNA seq reveals cell types in bladder tissue samples

Following single-cell suspension preparation with confirmed viability >85 %, the control and CYP groups yielded averages of (8.5 ± 1.2)×10^5^ and (7.8 ± 1.5) × 10^5^ viable cells per sample, respectively. Sequencing on the 10× Genomics platform captured an average of (9,500 ± 1,200) cells per sample, with no significant difference in capture efficiency between groups (p>0.05). After strict quality control, (8,200 ± 1,000) high-quality cells per sample were retained for analysis, with mitochondrial gene content consistently below 10 %.

**Figure 3:**
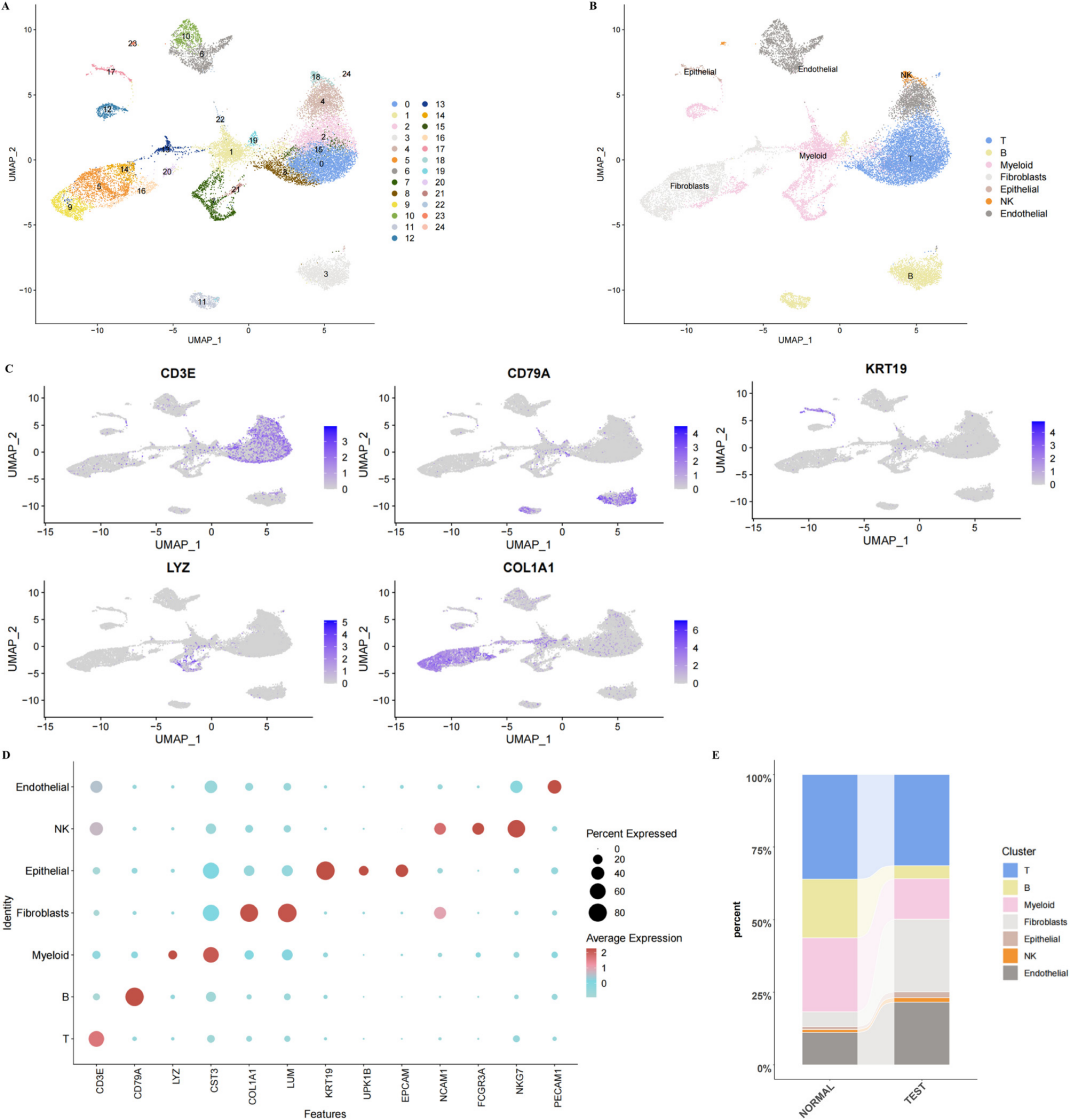
Single-cell annotation and cell clustering. Note: (A) UMAP projection derived from transcriptional profiles, revealing distinct cell clusters. (B) Annotation of cell identities in the UMAP space using established markers: T cells (CD3E), B cells (CD79A), myeloid cells (LYZ), fibroblasts (COL1A1), and epithelial cells (KRT19). (C) UMAP plots visualizing the expression distribution and level of individual marker genes. (D) dot plot quantifying the expression of feature genes across annotated cell types. The size of the dot encodes the percentage of cells expressing the gene, and the color encodes the average expression level in expressing cells. (E) Stacked bar plot displaying the relative frequency of each cell cluster across experimental conditions (NORMAL, n=8,300; TEST, n=8,100).

After strict quality control filtering to remove low-quality cells, and integration and clustering dimensionality reduction of the samples, a total of 25 cell clusters were identified (Figure 3A). Based on the expression of typical marker genes, the cells were manually annotated, and all cells in the bladder tissue sample were divided into the following cell types: T cells and B cells, myeloid cells, fibroblasts, NK cells, endothelial cells, and epithelial cells (Figure 3B and C). The relative expression levels of marker genes in each subgroup are shown in Figure 3D. In addition, single-cell sequencing results showed that the proportion of epithelial cells in the CYP group was significantly higher than that in the control group (Figure 3E). Therefore, we consider using epithelial cells as subsequent analysis cells.

#### Inter-cellular communication before and after IC disease

**Figure 4:**
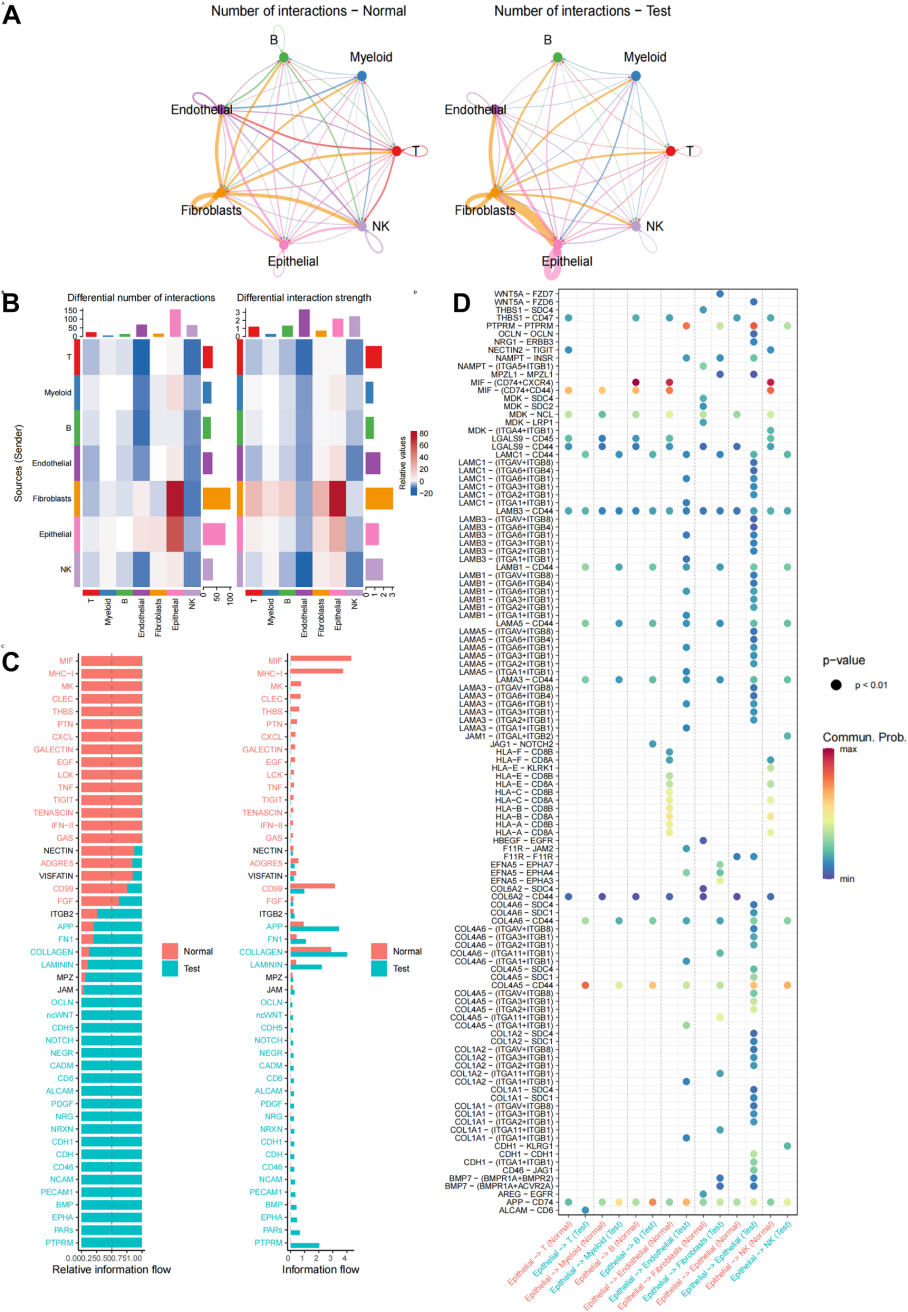
Analysis of intercellular communication. Note: (A) Circular layout of the cell-cell communication network. Outer node size corresponds to the cellular abundance of each cell type; colored nodes indicate sender cells. The width of the arched links is proportional to the number of inferred interactions between sender and receiver cell types. (B) Pairwise interaction analysis in the CYP group. The heatmaps quantify the total number of interactions (left) and the aggregate communication probability (right) between cell types. (C) Comparative analysis of overall signaling pathway activity. Bar plots illustrate the differences in information flow for each signaling pathway between groups, presented as relative changes (left) and absolute values (right). (D) specific ligand-receptor mediation analysis. The plot visualizes key mediated interactions between cell pairs (x-axis) and ligand-receptor pairs (y-axis). The color gradient of the circles denotes the communication probability, while the circle size is proportional to the statistical significance (-log10(p-value)).

Compared with the control group, the interaction between CYP-composing fibroblasts as sending cells and homologous or heterotypic cells is more extensive and close, with a higher probability of interaction (communication probability), while the possibility of T cells, B cells, and NK cells acting as sending cells on homologous or heterotypic cells is very small. From the perspective of the recipient, epithelial cells and fibroblasts act more as ligand-receptor interactions between receiving cells and homologous or heterotypic cells, while other cells have fewer or fewer interactions. It is worth noting that, both as sending cells and receiving cells, there are significantly more ligand receptor interactions between epithelial cells and various types of cells (Figure 4A and B). By comparing the information flow between cells and epithelial cells in the control group and CYP group, we noticed prominent signaling pathways in the CYP group, including collagen, amyloid precursor protein, laminin, and protein tyrosine phosphatase receptor M (PTPRM) (Figure 4C). In addition, by comparing the expression of ligand receptors between cells and epithelial cells in the control group and CYP group, we found that PTPRM-PTPRM was more prominently expressed in the CYP group, while MIF – (CD74 + CXCR4) was more prominently expressed in the control group, and both showed higher expression levels in specific cell populations or subgroups (Figure 4D).

#### DEG analysis of bladder tissue cells before and after IC disease

**Figure 5:**
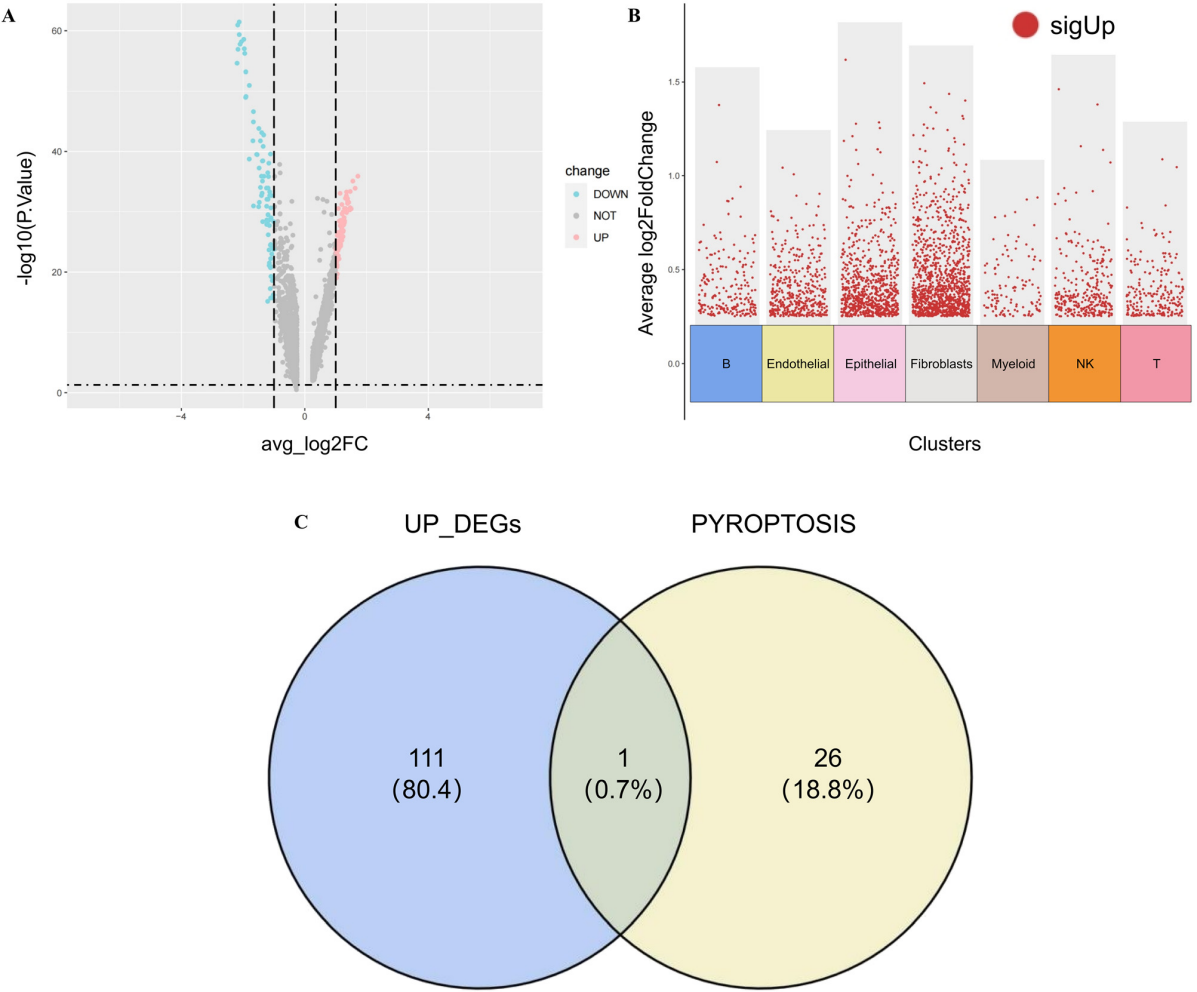
Core gene screening. Note: (A) Volcano plot visualizing genome-wide expression changes. Genes meeting the significance criteria (|log2FC|>1, adj. p<0.05) are colored (downregulated: Blue; upregulated: Pink). The gray points represent genes with non-significant alterations. (B) Violin plot with overlaid data points showing the expression pattern of upregulated DEGs across annotated cell clusters. The gray bars denote the mean expression level per cluster, while red dots show the expression in individual cells. (C) Venn diagram showing the significant intersection between upregulated DEGs in the CYP group and a predefined gene set of apoptosis-related factors.

By comparing the expression levels of each gene in bladder tissue cells, a total of 3,502 differentially expressed genes (DEGs) were selected. Among them, 112 genes were upregulated in the CYP group compared to the control group (LogFC>1), and 78 genes were downregulated in the CYP group compared to the control group (LogFC<−1). Further, the differential expression of genes was visually presented through volcano plots (Figure 5A). It was found that genes DHFR, CCSER1, PTPRM, COL4A5, and NAALADL2 were significantly upregulated in the CYP group (p<0.05), while genes RPS4X, IGLC2, IGHG3, RPL32, and S100A11 were significantly downregulated in the CYP group (p<0.05). The distribution of upregulated genes in each cell population is shown in Figure 5B, and upregulated genes are significantly enriched in epithelial cells (p<0.05).

#### Venn visualization for obtaining hub genes

Export 112 upregulated genes from DEGs and draw a Venn diagram (Figure 5C) by intersecting with 27 cell apoptosis-related genes. Select one Hub gene: TP63. This gene may be involved in the occurrence and development of IC by causing necrosis of bladder epithelial cells, leading to impaired bladder barrier function.

#### Enrichment analysis of gene expression heterogeneity in bladder tissue cells

**Figure 6:**
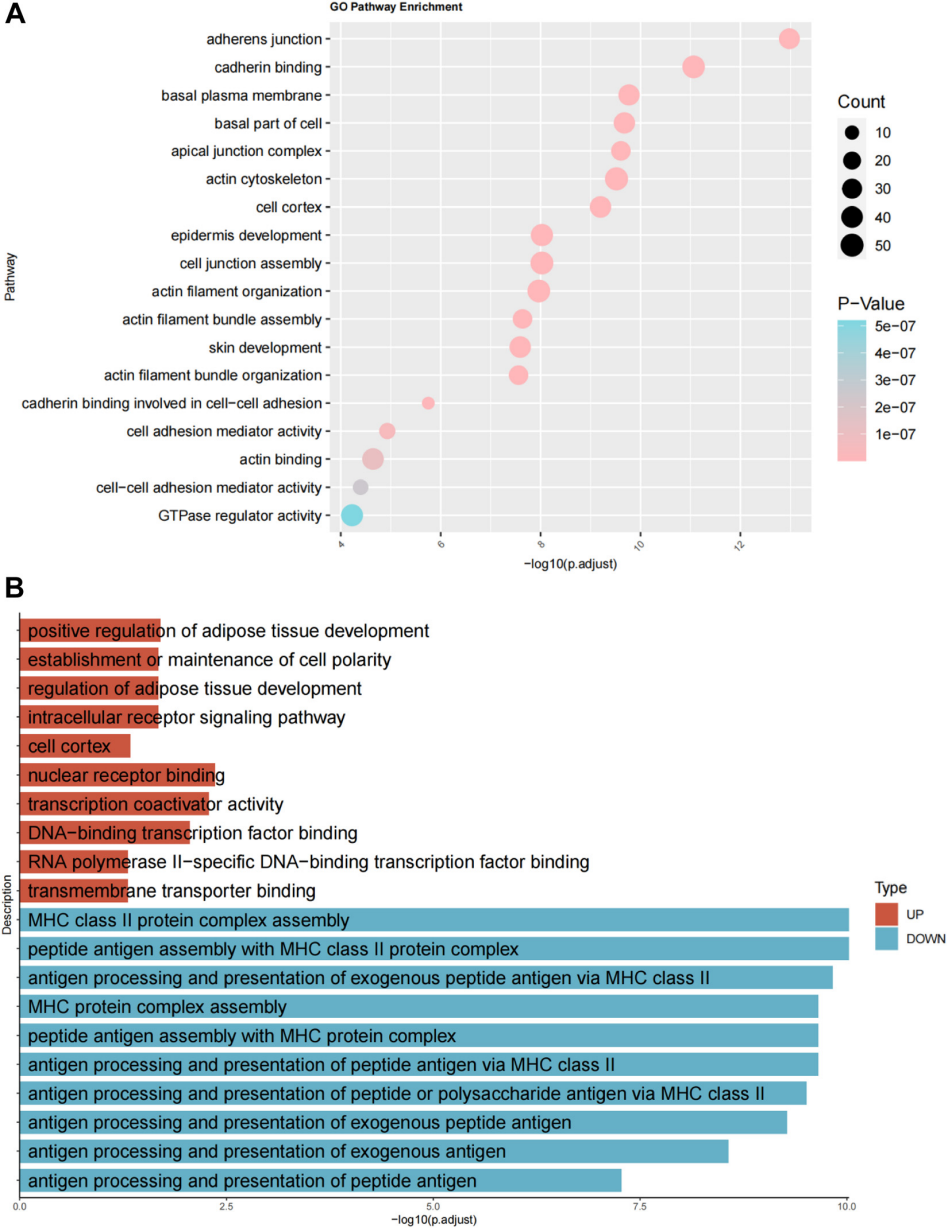
Visualization results of DEGs enrichment analysis. Note: (A) Bubble plot of significantly enriched gene ontology (GO) terms. The x-axis represents the enrichment significance [-log10 (adjusted p-value)]. The y-axis lists the specific GO terms. The size of the bubbles is proportional to the number of DEGs mapped to the corresponding term, and the color gradient reflects the adjusted p-value, with a darker color indicating higher statistical significance. (B) Horizontal bar graph of enriched kyoto encyclopedia of genes and genomes (KEGG) pathways. The x-axis indicates the enrichment significance [-log10 (adjusted p-value)]. The bars are colored to distinguish between pathways predominantly enriched for upregulated genes (red) and those enriched for downregulated genes (blue).

Perform GO functional and KEGG pathway enrichment analysis on differentially expressed genes in each subgroup to determine their potential biological functions. As shown in Figure 6A, after completing the biological process analysis of GO enrichment analysis, the top 18 most significantly enriched terms were selected using FDR value sorting. The results indicate that the biological functions of DEGs mainly focus on cell connectivity and adhesion, cell morphology and motility, cell division and differentiation, and cell signal transduction. The KEGG analysis results showed that significantly upregulated DEGs were enriched in pathways such as positive regulation of adipose tissue development, establishment and maintenance of cell polarity, while significantly downregulated DEGs were enriched in pathways such as assembly of MHC class II molecular complexes and assembly of peptide antigens and MHC class II protein complexes. Figure 6B shows the top 10 upregulated and downregulated pathways with enrichment scores, and differentially expressed genes may participate in the occurrence and development of IC through the aforementioned biological mechanisms.

### TP63 is highly expressed in both *in vivo* and *in vitro* IC models and inhibits cell viability and proliferation

#### High expression of TP63 in IC rat model

**Figure 7:**
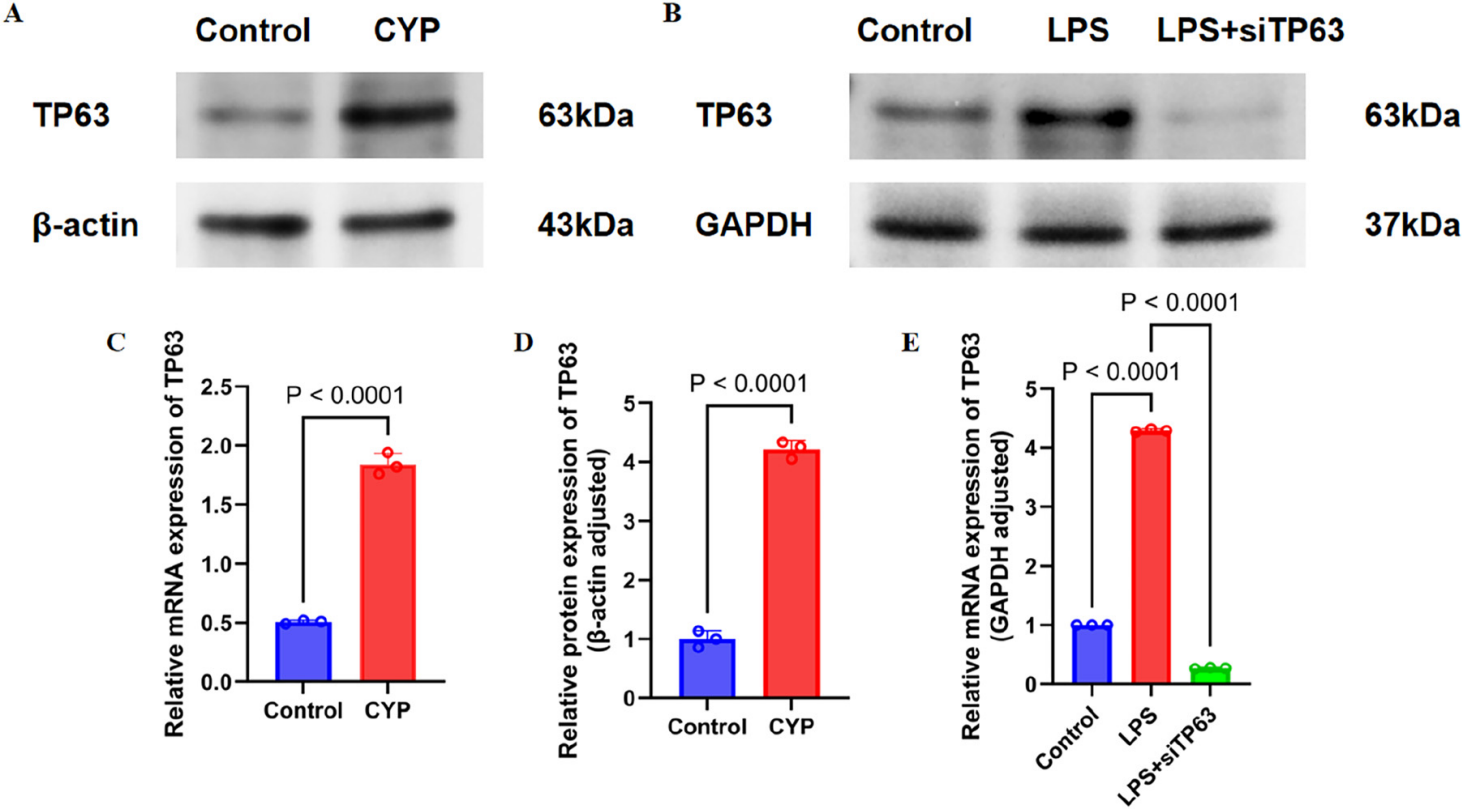
TP63 expression level detected by RT-PCR and western blot. Note: (A) Representative images of TP63 protein expression in bladder tissues from normal rats and CYP-induced rats. (B) Representative blot of TP63 protein expression in normal SV-HUC-1 cells and LPS-treated cells. (C) Quantitative analysis of TP63 mRNA levels in bladder tissues from normal and CYP-induced rats (n=3). Data are presented as M ± SD *p<0.0001, compared to the normal group by unpaired Student’s t-test. (D) statistical analysis of TP63 protein expression in bladder tissues from normal and CYP-induced rats (n=3). Data are presented as M ± SD *p<0.0001, compared to the normal group by unpaired Student’s t-test. (E) statistical analysis of TP63 protein expression in normal and LPS-treated SV-HUC-1 cells (n=3). Data are presented as M ± SD *p<0.0001, compared to the control group by unpaired Student’s t-test.

The Western blot analysis results showed that compared with normal rats, the expression level of TP63 protein in the CYP group rat bladder cell line was significantly upregulated (p<0.0001) (Figure 7A and D). The RT-PCR results also showed that compared to normal rats, the relative expression level of the TP63 gene mRNA was significantly upregulated in the CYP group rats (p<0.0001) (Figure 7C).

#### High expression of TP63 in SV-HUC-1 cell inflammatory model

As shown in Figure 7B and E, Western blot analysis showed that compared to normal SV-HUC-1 cells, the expression level of TP63 protein in the cells significantly increased after LPS treatment (p<0.0001).

#### The effect of TP63 expression levels on the viability of SV-HUC-1 cells

**Figure 8:**
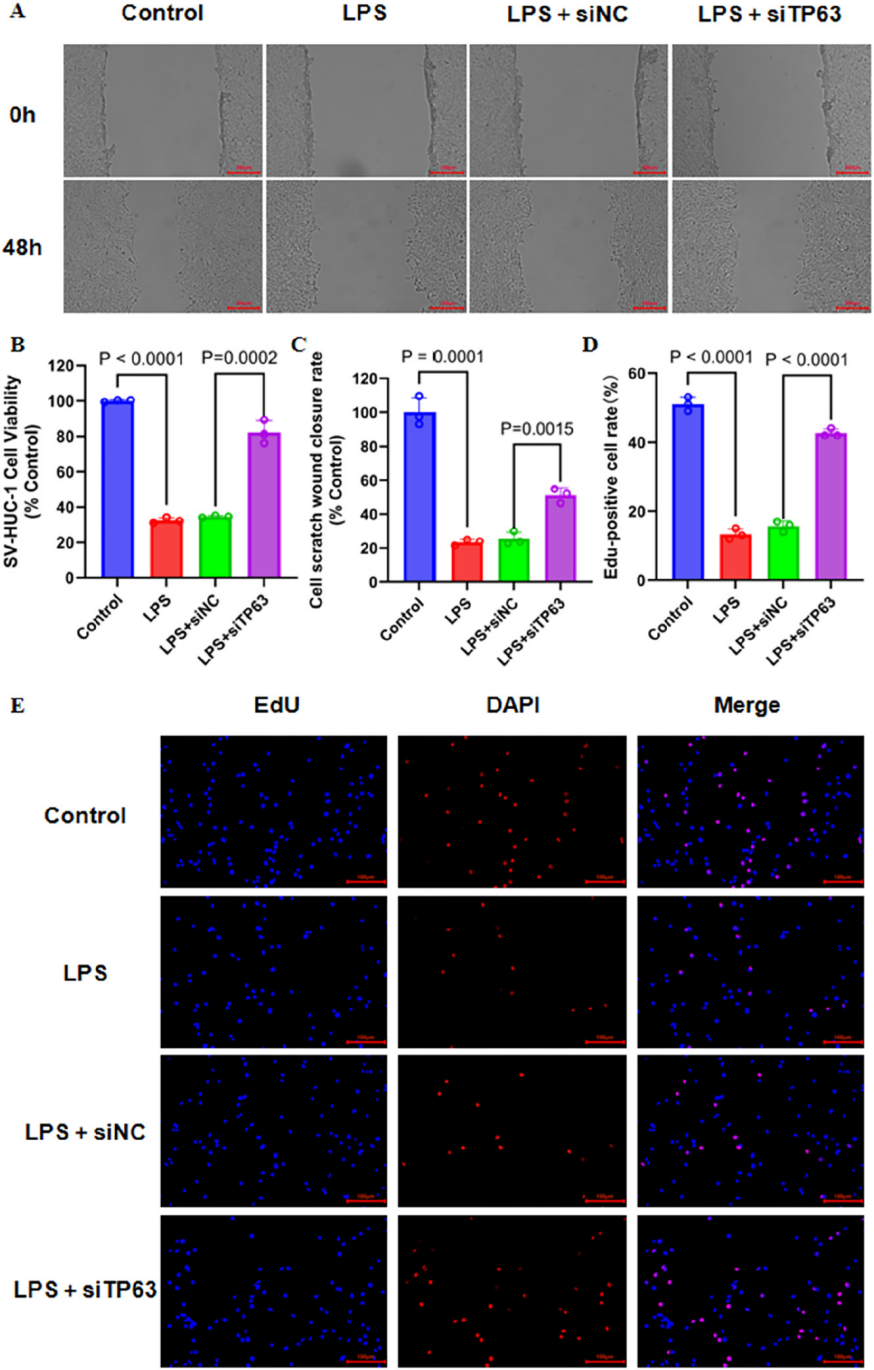
LPS inhibits SV-HUC-1 cell migration, viability and proliferation via the TP63 pathway. Note: (A, C) cell migration capacity evaluated by scratch wound healing assay. (A) Representative images of scratch closure (scale: 200 μm). (C) Quantitative analysis of wound closure rate (n=3). Data are presented as M ± SD (one-way ANOVA). (B) Cell viability measured by CCK-8 assay (n=3). Data are presented as mean ± SEM (one-way ANOVA). (D, E) cell proliferation assessed by EdU assay. (D) Representative fluorescence images (Edu and Hoechst 33,342 staining, x 400). (E) Quantitative analysis of EdU-positive cells (n=3). Data are presented as M ± SD (one-way ANOVA).

Compared with the control group, the SV-HUC-1 cell viability in the LPS-treated group was significantly reduced (p<0.0001), indicating the success of the LPS-induced SV-HUC-1 cell inflammation model. After LPS treatment, the TP63 gene of SV-HUC-1 cells was specifically knocked down, and cell viability was significantly increased (p<0.001) (Figure 8B). These results reveal that LPS has an inhibitory effect on SV-HUC-1 cell viability, and the expression of the TP63 gene also has an inhibitory effect on SV-HUC-1 cell viability.

#### The effect of TP63 expression level on the migration ability of SV-HUC-1 cells

As shown in Figure 8A and C, the LPS treatment group showed a lower cell scratch healing rate compared to the control group (p<0.001). Furthermore, compared with the LPS + siNC group, the LPS + siTP63 group showed a significant improvement in cell scratch healing rate (p<0.01). These data indicate that LPS can inhibit the migration of SV-HUC-1 cells, while specific knockdown of TP63 helps to reverse the migration effect caused by LPS.

#### The effect of TP63 expression level on the proliferation ability of SV-HUC-1 cells

As shown in Figure 8D and E, compared with the control group, the EdU positive rate of cells in the LPS treatment group was significantly reduced (p<0.01). After TP63 siRNA intervention, the positivity rate of EdU significantly increased (p<0.05). These results reveal that LPS has an inhibitory effect on SV-HUC-1 cell proliferation, while TP63 siRNA intervention can promote LPS-induced SV-HUC-1 cell proliferation.

### TP63 exerts a regulatory effect by activating the NF-κB signaling pathway

To investigate whether TP63 directly regulates the NF-κB signaling pathway, we performed ChIP-qPCR analysis to assess the binding of TP63 to the promoter region of RELA, a key subunit of the NF-κB complex. The results demonstrated that TP63 binds to the RELA promoter under various treatment conditions (Figure 10A). Quantitative analysis from three independent experiments (n=3) revealed a significant increase in TP63 binding to the RELA promoter in LPS-treated cells compared with control cells, with a fold enrichment exceeding 30 (p<0.0001). These findings indicate that TP63 directly interacts with the RELA promoter, suggesting a mechanism by which TP63 may activate the NF-κB signaling pathway to exert its regulatory functions.

### TP63 induces pyroptosis through the NF-κB/NLRP3/caspase-1 axis

#### TP63 causes upregulation of apoptosis-related proteins

**Figure 9:**
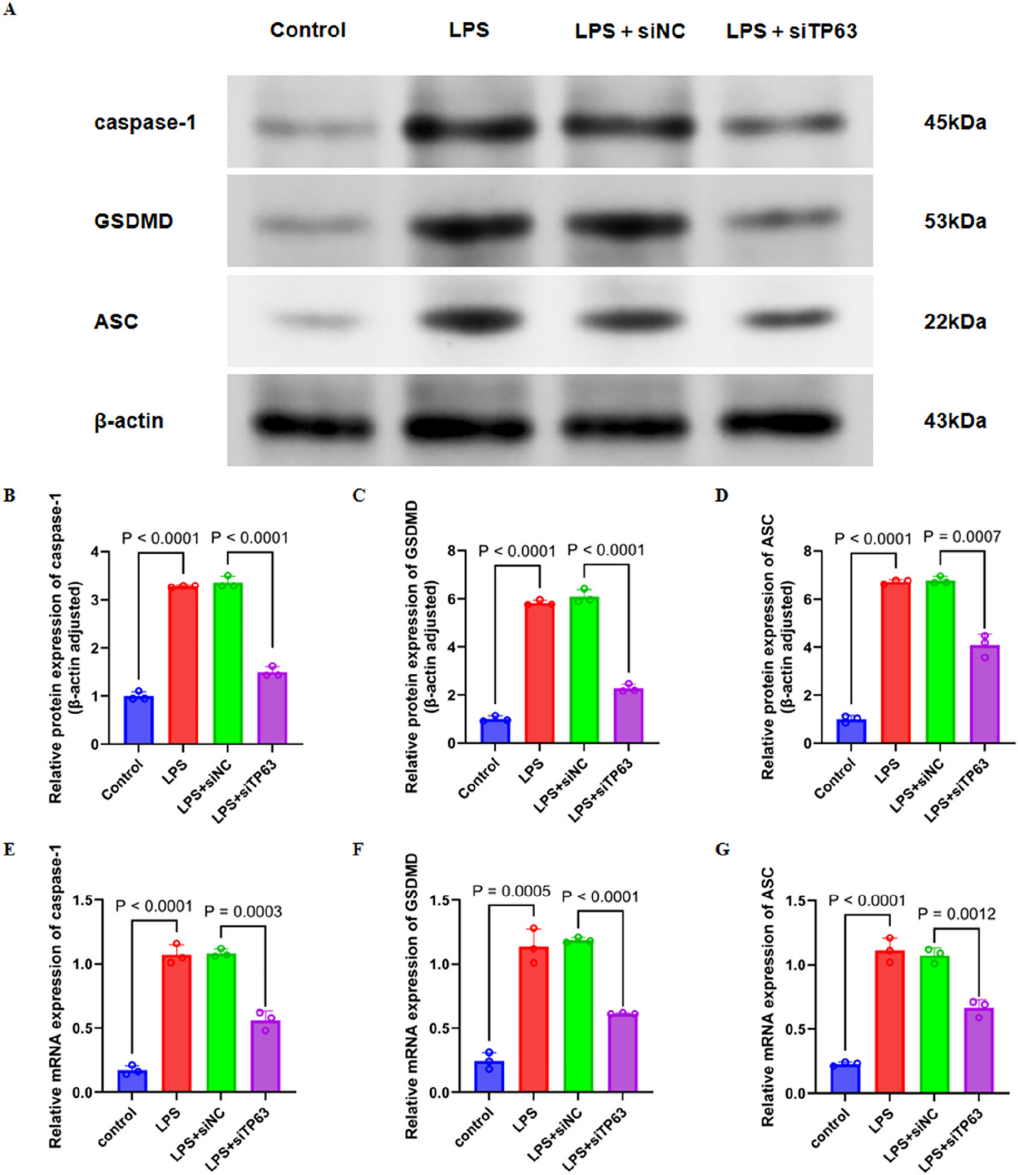
Effects of LPS and TP63 siRNA on apoptosis-related gene and protein expression in SV-HUC-1 cells. Note: (A) Representative western blot images of TP63 and apoptosis-related proteins in SV-HUC-1 cells from control, LPS-treated, and LPS + TP63 siRNA groups. (B–D) mRNA expression levels of apoptosis-related genes in SV-HUC-1 cells among different groups (n=3). Data are presented as M ± SD (one-way ANOVA with Tukey’s post hoc test). (E–G) Quantitative analysis of apoptosis-related protein expression levels in SV-HUC-1 cells among different groups (n=3). Data are presented as M ± SD (one-way ANOVA with Tukey’s post hoc test).

Based on the finding that TP63 binds to the RELA promoter and activates the NF-κB signaling pathway, we further investigated whether TP63 regulates pyroptosis through the NF-κB/NLRP3/caspase-1 axis. The RT-qPCR results demonstrated that LPS treatment significantly upregulated the mRNA expression of key pyroptosis-related genes, including caspase-1, GSDMD, and ASC, compared to the normal control group (p<0.0001, Figure 9B–D). Importantly, TP63 knockdown markedly attenuated this effect, with significant reductions in the expression of caspase-1 (p<0.0001), GSDMD (p<0.0001), and ASC (p<0.001) compared to the LPS + siNC group.

Western blot analysis consistently revealed corresponding changes at the protein level (Figure 9A, E–G), confirming the regulatory role of TP63 in the NF-κB/NLRP3/caspase-1 pathway. Our findings demonstrate that TP63 induces pyroptosis in SV-HUC-1 cells through a defined molecular cascade: the binding of TP63 to the RELA promoter activates the NF-κB pathway, which then triggers the NLRP3/caspase-1 axis to upregulate key executioners like GSDMD.

#### Caspase-1 inhibition experiment

**Figure 10:**
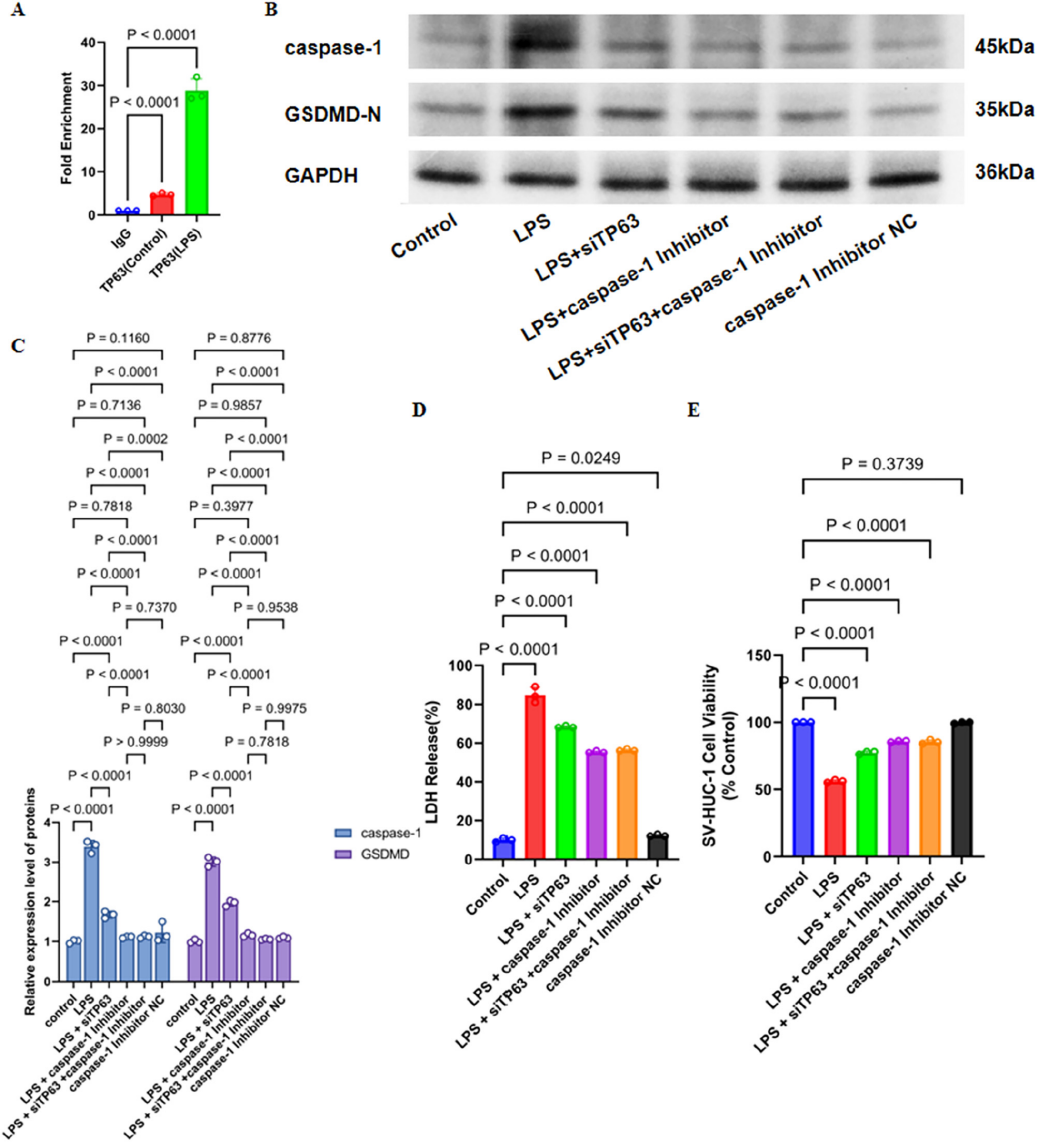
TP63 binds to the RELA promoter and regulates caspase-1-mediated pyroptosis in SV-HUC-1 cells. Note: (A) ChIP-qPCR analysis of TP63 binding to the RELA promoter under different treatment conditions (n=3). Data are presented as mean ± SD (one-way ANOVA with Tukey’s post hoc test). (B) Representative western blot images showing protein expression of caspase-1 and GSDMD-N in different treatment groups. GAPDH was used as a loading control. (C) Quantitative analysis of caspase-1 and GSDMD-N protein expression normalized to GAPDH (n=3). Data are presented as M ± SD (one-way ANOVA with Tukey’s post hoc test). (D) pyroptosis level assessed by LDH release assay (n=3). Data are presented as M ± SD (one-way ANOVA with Tukey’s post hoc test). (E) cell viability measured by CCK-8 assay (n=3). Data are presented as M ± SD (one-way ANOVA with Tukey’s post hoc test).

Western blot analysis demonstrated that LPS treatment significantly upregulated the expression of both caspase-1 and its substrate GSDMD-N compared to control cells (p<0.0001, Figure 10B and C). Notably, this effect was substantially reversed by either caspase-1 inhibition or TP63 knockdown, indicating that TP63 is essential for caspase-1 activation and subsequent GSDMD cleavage.

To confirm the functional consequences of these molecular events, we performed LDH release and CCK-8 assays. The LDH release assay revealed that LPS treatment induced significant pyroptosis, which was markedly attenuated by either caspase-1 inhibition or TP63 knockdown (p<0.0001, Figure 10D). Correspondingly, the CCK-8 assay showed that the decreased cell viability caused by LPS was partially restored by interfering with either caspase-1 or TP63 (p<0.0001, Figure 10E).

Collectively, these results demonstrate that pyroptosis in SV-HUC-1 cells is ultimately induced through a cascade where TP63 binding to the RELA promoter activates the NF-κB pathway, which then triggers the NLRP3/caspase-1 axis and leads to GSDMD cleavage.

## Discussion

This study systematically clarifies the critical function of the TP63 gene in interstitial cystitis (IC) pathogenesis. TP63 mediates pyroptosis via the NF-κB/NLRP3/caspase-1 signaling axis. These findings are supported by an integrated approach combining single-cell sequencing and multi-omics validation.

Although research on IC pathogenesis is not uncommon, its etiology and pathophysiology remain incompletely understood. Recent breakthroughs in single-cell sequencing have opened new avenues for analyzing complex disease landscapes [13]. In this work, single-cell transcriptome sequencing was used and revealed – for the first time – marked heterogeneity in bladder epithelial cells from IC specimens. UMAP clustering showed that the proportion of bladder epithelial cells was substantially increased in the CYP group compared to controls. Cell communication analysis further confirmed significantly enhanced ligand–receptor interactions between epithelial cells and other cell types. These observations align with earlier clinical reports [14], reinforcing the indispensable role of bladder epithelial cells in IC progression [15].

In terms of experimental models, the CYP-induced interstitial cystitis rat model and the LPS-stimulated SV-HUC-1 cell model used in this study proved to be highly applicable. Both models effectively recapitulated the core pathophysiological features of interstitial cystitis. The CYP model reliably reproduced key clinical manifestations. For instance, it showed urodynamic abnormalities – including shortened voiding intervals and elevated maximum detrusor pressure – which mirror the urinary frequency and urgency reported in patients. The model also displayed histopathological alterations, such as bladder wall edema, inflammatory infiltration, and epithelial injury, thus simulating the characteristic inflammatory response and tissue remodeling seen in interstitial cystitis. Increased pain sensitivity, a hallmark of the disease, was further confirmed via Von-Frey testing. Meanwhile, the LPS-induced SV-HUC-1 cell model successfully replicated essential cellular events observed in interstitial cystitis. These included epithelial barrier dysfunction, inflammatory cytokine release, and pyroptosis. While these models do not capture the full complexity of human interstitial cystitis, they hold clear scientific value in modeling its central pathological processes.

Notably, this study identified significant upregulation of TP63 in the bladder tissue of interstitial cystitis rats. This abnormal expression was mainly localized to bladder epithelial cells. This finding stands in striking contrast to the widely reported role of TP63 in maintaining mucosal barrier integrity [16], 17]. We speculate that this discrepancy may be closely related to the isoform heterogeneity of TP63 [18], 19]. Previous studies have highlighted ΔNp63α as a key factor in preserving the undifferentiated state of epithelial cells. Its downregulation leads to excessive basal cell proliferation without proper differentiation into superficial cells, ultimately disrupting epithelial architecture and weakening barrier function [20]. However, what we detected may represent another isoform – TAp63. Its upregulation could induce aberrant cell death or senescence [21], 22], similarly compromising barrier integrity. Barrier defects facilitate local inflammatory infiltration and nerve fiber proliferation. These changes, in turn, inflict further damage on epithelial cells through oxidative stress and neurogenic inflammation. This establishes a self-sustaining “barrier disruption–inflammation amplification” cycle that drives the chronicity of interstitial cystitis. Another possibility is that our single-cell sequencing focused on rapidly progressing or fibrotic subtype SD rats. In these animals, elevated TP63 expression may upregulate TGF-β1 and collagen synthase as part of a compensatory repair program, thereby promoting bladder wall fibrosis [23].

At the molecular mechanistic level, this study provides direct evidence supporting the hypothesis that “TP63 regulates cellular pyroptosis via the NF-κB/NLRP3/caspase-1 signaling axis” through multi-layered experimental data. First, ChIP-qPCR analysis demonstrated that TP63 can directly bind to the promoter region of RELA (p65), a core transcription factor of the NF-κB pathway, thereby establishing the biological relationship of TP63 as an upstream regulator of NF-κB signaling at the transcriptional level. Second, functional experiments further confirmed the regulatory role of TP63 in the execution phase of pyroptosis: in a lipopolysaccharide-induced cellular inflammation model, knockdown of TP63 expression using siRNA significantly inhibited the activation of caspase-1 and the cleavage of its substrate GSDMD, while concurrently reversing the LPS-induced decline in cell viability and the increase in pyroptosis-associated LDH release. These consistent results from both *in vivo* and *in vitro* models collectively form a coherent chain of evidence, indicating that TP63 is a key upstream regulator activating caspase-1-dependent pyroptosis in bladder epithelial cells.

Based on the aforementioned direct experimental evidence and integrating the widely accepted mechanistic framework in this field – namely, that activation of the NF-κB signaling pathway is a critical step in priming the transcription of the NLRP3 inflammasome and related pro-inflammatory cytokines (e.g., pro-IL-1β) [24], 25] – this study further synthesizes and proposes a cascade regulatory model: “TP63→RELA/NF-κB→NLRP3 inflammasome→caspase-1→GSDMD-mediated pyroptosis.” We observed that under conditions of elevated TP63 expression, protein levels of NLRP3 inflammasome components (e.g., ASC) and its downstream effector molecule GSDMD were correspondingly increased, consistent with the core predictions of this model. However, we also objectively note that this study has not yet completed the final validation of all connecting steps within this model. For instance, whether TP63 binding to the RELA promoter directly drives p65 protein phosphorylation, nuclear translocation, and the transcriptional activity of downstream target genes requires further supporting evidence. The process by which NLRP3 and ASC form a functional inflammasome complex within cells also awaits direct observation via methods such as immunofluorescence co-localization. Furthermore, the release levels of typical terminal products of pyroptosis – mature IL-1β and IL-18 – were not quantitatively assessed in this study. In-depth validation of these aspects represents an important direction for future research to refine this mechanistic map.

In summary, we propose the following mechanism in the pathological progression of interstitial cystitis. Specifically, TP63 (particularly the TAp63 isoform) directly binds to and transcriptionally activates the RELA promoter, thereby initiating the NF-κB signaling pathway. Activated NF-κB subsequently upregulates the expression of the NLRP3 inflammasome, which promotes caspase-1 activation and GSDMD cleavage. This cascade ultimately induces pyroptosis in bladder epithelial cells. The inflammatory mediators released during pyroptosis further amplify local neuroimmune responses, establishing a self-perpetuating “epithelial damage–inflammation cycle.” The resulting sustained inflammatory microenvironment activates pro-fibrotic pathways such as TGF-β1, driving tissue remodeling and fibrosis of the bladder wall. This study directly demonstrates that TP63 functionally regulates NF-κB transcription as well as the caspase-1/GSDMD-dependent pyroptosis axis. These findings connect TP63-mediated transcriptional regulation, NF-κB-initiated inflammation, pyroptotic execution, and downstream fibrosis into a logically coherent cascade. Together, they provide an integrated mechanistic perspective for understanding the progression of interstitial cystitis from programmed epithelial cell death to chronic organ fibrosis.

This study has several limitations. Although the experimental models are widely accepted, they still fall short of fully capturing the complexity of human IC. Due to a lack of reliable isoform-specific antibodies, we could not distinguish between TP63 isoforms. Time constraints also prevented the completion of TP63 overexpression rescue experiments. Our current focus has been primarily on the NF-κB/NLRP3/caspase-1 axis, leaving other potential pathways unexplored. Moreover, all animal experiments used female rats, so the influence of sex differences remains unassessed.

Based on our findings, TP63-targeted therapy demonstrates considerable clinical translational potential. The TP63 expression profile could serve as a biomarker for IC molecular subtyping, guiding personalized treatment: patients with high TAp63 expression may respond better to NLRP3 inhibitors (e.g., VX-765) or caspase-1 inhibitors, whereas those with low ΔNp63 might benefit from epithelial repair-promoting agents. Developing specific modulators targeting different TP63 isoforms also holds promise – TAp63 inhibitors could temper inflammatory responses, while ΔNp63 agonists may facilitate barrier restoration. Furthermore, TP63 expression levels might help predict therapeutic efficacy, identifying patient subgroups most likely to respond to targeted interventions. These approaches open new paths for devising precision medicine strategies in IC. Future efforts should prioritize preclinical validation and translational exploration to advance TP63-targeted therapies from bench to bedside.

Subsequent research should delve deeper into the specific regulatory mechanisms of TP63 isoforms and validate these insights in animal models of both sexes and clinical samples, thereby strengthening the scientific foundation for precision medicine in IC.

## Conclusions

Based on single-cell sequencing analysis, we demonstrated the critical role of the TP63 gene in interstitial cystitis. Our findings indicate that TP63 promotes a specific form of cell death – pyroptosis – in the bladder lining through a signaling cascade involving NF-κB, NLRP3, and caspase-1.

This study further reveals that the regulatory effect of TP63 on pyroptosis is most pronounced in bladder epithelial cells. The core mechanism involves the direct binding of TP63 to the NF-κB pathway and its subsequent transcriptional activation, which initiates a downstream signaling cascade that ultimately executes pyroptosis.

These results advance our understanding of disease pathogenesis. They suggest that assessing TP63 expression levels could aid in clinical diagnosis, and that different TP63 gene subtypes or variants may require tailored treatment strategies. Together, these insights point toward potential future directions for personalized precision medicine in this condition.

Future research should test these findings in human patients. Scientists should also work on developing treatments that target the specific TP63 versions involved in this disease.
